# Serum Levels of Soluble CD26/Dipeptidyl Peptidase-IV in Type 2 Diabetes Mellitus and Its Association with Metabolic Syndrome and Therapy with Antidiabetic Agents in Malaysian Subjects

**DOI:** 10.1371/journal.pone.0140618

**Published:** 2015-10-16

**Authors:** Radwan H. Ahmed, Hasniza Zaman Huri, Zaid Al-Hamodi, Sameer D. Salem, Sekaran Muniandy

**Affiliations:** 1 Department of Molecular Medicine, Faculty of Medicine, University of Malaya, Kuala Lumpur, Malaysia; 2 Department of Pharmacy, Faculty of Medicine, University of Malaya, Kuala Lumpur, Malaysia; 3 Clinical Investigation Centre, University Malaya Medical Centre, Kuala Lumpur, Malaysia; 4 Department of Biochemistry and Molecular Biology, Faculty of Medicine, Sana’a University, Sana’a, Yemen; University of Leuven, Rega Institute, BELGIUM

## Abstract

**Background:**

A soluble form of CD26/dipeptidyl peptidase-IV (sCD26/DPP-IV) induces DPP-IV enzymatic activity that degrades incretin. We investigated fasting serum levels of sCD26/DPP-IV and active glucagon-like peptide-1 (GLP-1) in Malaysian patients with type 2 diabetes mellitus (T2DM) with and without metabolic syndrome (MetS), as well as the associations between sCD26/DPP-IV levels, MetS, and antidiabetic therapy.

**Methods:**

We assessed sCD26/DPP-IV levels, active GLP-1 levels, body mass index (BMI), glucose, insulin, A1c, glucose homeostasis indices, and lipid profiles in 549 Malaysian subjects (including 257 T2DM patients with MetS, 57 T2DM patients without MetS, 71 non-diabetics with MetS, and 164 control subjects without diabetes or metabolic syndrome).

**Results:**

Fasting serum levels of sCD26/DPP-IV were significantly higher in T2DM patients with and without MetS than in normal subjects. Likewise, sCD26/DPP-IV levels were significantly higher in patients with T2DM and MetS than in non-diabetic patients with MetS. However, active GLP-1 levels were significantly lower in T2DM patients both with and without MetS than in normal subjects. In T2DM subjects, sCD26/DPP-IV levels were associated with significantly higher A1c levels, but were significantly lower in patients using monotherapy with metformin. In addition, no significant differences in sCD26/DPP-IV levels were found between diabetic subjects with and without MetS. Furthermore, sCD26/DPP-IV levels were negatively correlated with active GLP-1 levels in T2DM patients both with and without MetS. In normal subjects, sCD26/DPP-IV levels were associated with increased BMI, cholesterol, and LDL-cholesterol (LDL-c) levels.

**Conclusion:**

Serum sCD26/DPP-IV levels increased in T2DM subjects with and without MetS. Active GLP-1 levels decreased in T2DM patients both with and without MetS. In addition, sCD26/DPP-IV levels were associated with Alc levels and negatively correlated with active GLP-1 levels. Moreover, metformin monotherapy was associated with reduced sCD26/DPP-IV levels. In normal subjects, sCD26/DPP-IV levels were associated with increased BMI, cholesterol, and LDL-c.

## Introduction

Diabetes mellitus is a heterogeneous group of disorders [1] that affected 387 million people worldwide in 2014 and this number is expected to rise to 592 million, or 10% of adults, by 2035 [2]. In 2014, the prevalence of diabetes mellitus among Malaysia’s adult population was reported to be 16.6% [2].

Dipeptidyl peptidase-IV (DPP-IV) is a serine protease found on the apical surface of different cells that cleaves X-proline dipeptides from the N-terminus of many polypeptides, including chemokines, peptide hormones, and neuropeptides [3]. In addition to its membrane form, DPP-IV is also found in a soluble form known as cell surface antigen CD26 (sCD26/DPP-IV). This soluble form is a found in a variety of biological fluids, and it originates when the transmembrane protein is shed [4]. Some studies have suggested that the soluble form of DPP-IV originates from adipocytes and immune cells [3, 5].

Glucose-dependent insulinotropic polypeptides (GIP) and glucagon-like peptides (GLP-1) are major incretin hormones that are rapidly inactivated by sCD26/DPP-IV. Hence, sCD26/DPP-IV has been studied intensively for the management of T2DM [6]. The soluble form of CD26 (sCD26/DPP-IV) causes DPP-IV enzymatic activity in the extracellular domain [7, 8].

Dysfunction of pancreatic β-cells, insulin resistance, and chronic low-grade inflammation are the main abnormalities associated with T2DM [9]. Adipose tissue macrophages are involved in the development of insulin resistance and chronic inflammation [10]. Previous studies have demonstrated that in the visceral adipose tissue of diet-induced diabetic mice, there was an increase in the numbers of CD8+ T-cells and CD11c+ M1 macrophages, and their infiltration into visceral adipose tissue was prevented by sCD26/DPP-IV inhibition, indicating that sCD26/DPP-IV might be implicated in the inflammation of adipose tissue [11]. In addition, it was found that sCD26/DPP-IV plays a role in regulating glycemia [12, 13]. Recently, it was reported that serum DPP-IV activity in type 1 diabetics (T1DM) was associated with insulin resistance [14]. In addition, an increase of DPP-IV activity in normal subjects is a predictor for MetS and insulin resistance and could be considered a novel biomarker for insulin resistance and MetS [15].

Nearly 90–95% of serum DPP-IV activity is related to sCD26/DPP-IV levels [8], a finding that is supported by several epidemiological studies [16–18]. However, Cordero et al. [4] reported that DPP-IV activity and sCD26/DPP-IV concentration are not always correlated.

A number of clinical studies have been conducted to demonstrate whether DPP-IV activity is associated with either the severity or onset of diabetes or with obesity. These studies showed controversial results, with DPP-IV activity either decreasing [19–21] or increasing [22, 23] in T2DM patients.

Despite the body of research in this area, little is known about the associations between fasting serum sCD26/DPP-IV levels, metabolic disorders, and T2DM. In addition, the associations between serum sCD26/DPP-IV levels in T2DM patients either with or without MetS are still unclear.

The aim of the current study was to evaluate fasting serum sCD26/DPP-IV levels and active GLP-1 levels in Malaysian T2DM patients with and without MetS, as well as to investigate the association of sCD26/DPP-IV level with MetS parameters and antidiabetic agents. In addition, we examined the correlation between serum sCD26/DPP-IV levels and active GLP-1 levels. Finally, this study also assessed the association between sCD26/DPP-IV levels and MetS parameters in normal subjects.

## Materials and Methods

### Subjects

This study involved diabetic and non-diabetic patients, both with and without MetS, who were diagnosed and treated at the Medical Centre, University of Malaya (UMMC). Normal subjects without either MetS or diabetes were enrolled as a control group and underwent a routine medical check-up. The study followed the principles set out in the Declaration of Helsinki and was conducted with approval by the Medical Ethics Committee of the University Malaya Medical Centre (approval number 387.15). Data were collected after obtaining written consent from each participant. Patients who had a malignancy, chronic or acute diseases of the liver, heart, or kidney, or who had received DPP-IV inhibitors were excluded from this study. The therapy regimens for diabetic patients using antidiabetic agents were obtained from patient records at UMMC.

### Anthropometric measurements

Blood pressure (BP) was measured in the morning 10 minutes after resting in a quiet room using an Omron IntelliSense Automatic Blood Pressure monitor. The average of three BP readings was recorded. The weight and height of each subject were measured in the morning after fasting for 12 hours. BMI was calculated as weight (kg) divided by height (m^2^), and waist circumference at the midpoint between the lowest rib and the frontal superior iliac spine was also measured. A 10 mL sample of fasting venous blood was taken from each participant.

### Measurements of serum sCD26/DPP-IV and active GLP-1 levels

Serum cholesterol (TC), triglyceride, HDL-c, and plasma glucose (FPG) levels were measured using an automated analyzer (Dimension® RxL Max®, Integrated Chemistry System). Fasting plasma insulin (FPI) levels were measured using the ADVIA Centaur XP Immunoassay System (Siemens Healthcare Diagnostics Inc., Deerfield, IL, USA), and the variant glycosylated hemoglobin (A1c) reorder pack (Catalogue number 270–0003) (Bio-Rad, USA) was used to measure A1c levels. Insulin resistance (HOMA-IR) and β-cell function (HOMA-β) were calculated using the Homeostasis Model Assessment (HOMA2) Calculator v2.2 (http://www.dtu.ox.ac.uk/homacalculator/index.php), according to procedures outlined by Matthews et al. [24]. Fasting serum levels of sCD26/DPP-IV were measured using the Human sCD26 ELISA Kit (RayBiotech, Inc., USA) according to the manufacturer’s instructions. Absorbance in 96-well plates was read at 450 nm with a microplate reader (Hydroflex Elisa, Chemopharm, Austria).

Serum active GLP-1 levels, including GLP-1 (7–36) and GLP-1 (7–37), were determined by ELISA (EMD, Millipore Inc., USA), which measures active GLP-1 without cross-reacting with other inactive forms of GLP-1. The analysis was performed manually according to the manufacturer’s instructions.

### Statistical analysis

Biostatistical analyses were performed using SPSS Package Version 11.5 (LEAD Technologies, Inc., USA). *P-*values < 0.05 were considered to be significant. Log_10_ transformation was performed for the standard biochemical and demographic parameters, including serum levels of sCD26/DPP-IV and active GLP-1, because they were not normally distributed. After back transformation, means were expressed as a geometric means and standard deviation (SD). The association between fasting serum levels of sCD26/DPP-IV and active GLP-1 in T2DM and non-T2DM patients, both with and without MetS, were studied after correcting for age, race, and gender as covariates by general linear model. Correlations between serum levels of sCD26/DPP-IV and active GLP-1 were assessed by the Spearman partial correlation coefficient (r_s_).

A multiple linear regression analysis was performed in diabetic patients to investigate the associations between serum levels of sCD26/DPP-IV (as the dependent variable) and metabolic parameters, A1c levels, and metformin therapy. Hierarchical linear regression was applied to evaluate the associations between serum levels of sCD26/DPP-IV and diabetic and metabolic parameters (as the dependent variable) in normal subjects.

## Results

### Fasting serum levels of sCD26/DPP-IV and active GLP-1 in the study groups

A total of 549 subjects were enrolled in this study. Patients were divided into study groups according to diagnoses by attending physicians and endocrinologists, who performed standard biochemical tests and applied the IDF criteria model for MetS diagnosis [25]. There were 164 control subjects without either MetS or diabetes mellitus and 71 non-diabetic participants with MetS who were under treatment for hypertension and/or hyperlipidemia. The study also included 314 subjects already diagnosed with T2DM; of these, 57 did not have MetS, and 257 had MetS. The basic demographics and biochemical parameters of the normal subjects, the non-diabetic patients with MetS, the diabetic patients with MetS, and the diabetics without MetS are summarized in (Table 1)

**Table 1:** Demographic and standard biochemical parameters among normal control, non-diabetes metabolic syndrome, type 2 diabetes mellitus subjects with and without metabolic syndrome.

	Non-diabetes n = 235			Type 2 diabetes n = 314			
Parameters	Normal Control n = 164(29.9%)	MetS n = 71(12.9%)	P-Value	Without MetS n = 57(10.4%)	P-Value	With MetS n = 257(46.8%)	P-Value
**Gender % (Male/Female)**	39.0/61.0	59.2/40.8		56.1/43.9		44.4/55.6	
**Race (Malay %)**	52.4	43.6		36.8		51.4	
**Race (Chinese %)**	31.7	28.2		26.3		14.8	
**Race (Indian %)**	15.9	28.2		36.9		33.8	
**Age (years)**	48.3(12.30)	50.9(9.12)	^a^ 0.443	50.0(8.91)	^a^ ^,^ ^*b*^ 0.999	50.7(7.59)	^a^ 0.123,^*b*^ ^,^ ^c^ 0.999
**Weight (kg)**	60.5(13.80)	74.0(13.80)	^a^ ***1*.*3×10*** ^***−10***^	62.6(13.49)	^a^ 0.999, ^*b*^ ***4*.*3×10*** ^***−5***^	72.1(15.14)	^a^ ***1*.*3×10*** ^***−15***^, ^*b*^0.999, ^c^ ***2*.*5×10*** ^***−5***^
**Height (m)**	1.61(0.09)	1.64(0.09)	^a^ *0*.*426*	1.60(0.10)	^a^ 0.999, ^*b*^ *0*.*273*	1.58(0.10)	^a^ ***0*.*005***, ^*b*^ ***7*.*6×10*** ^***−5***^, ^c^0.660
**Body Mass Index (kg/m** ^**2**^ **)**	23.3(4.27)	27.6(4.68)	^a^ ***3*.*2×10*** ^***−10***^	24.3(4.68)	^a^ *0*.*765*, ^*b*^ ***3*.*8×10*** ^***−4***^	28.9(5.25)	^a^ ***3*.*8×10*** ^***−29***^, ^*b*^ *0*.*344*, ^c^ ***4*.*5×10*** ^***−10***^
**Waist Circumference (cm)**	81.5(12.3)	94.9(10.72)	^a^ ***1*.*01×10*** ^***−14***^	86.0(12.30)	^a^ *0*.*047*, ^*b*^ ***1*.*5x10*** ^***-4***^	96.6(11.48)	^a^ ***2*.*3×10*** ^***−33***^, ^*b*^0.999, ^c^ ***1*.*1×10*** ^***−8***^
**Systolic Blood Pressure (mmHg)**	130(19.50)	143(15.14)	^a^ ***1*.*3×10*** ^***−5***^	120(16.60)	^a^ ***0*.*001*,** ^*b*^ ***4*.*02×10*** ^***−11***^	138(18.62)	^a^ ***1*.*7×10*** ^***−4***^, ^*b*^ *0*.*315*, ^c^ ***2*.*1×10*** ^***−10***^
**Diastolic Blood Pressure (mmHg)**	81(9.77)	86(8.91)	^a^ ***3*.*5×10*** ^***−4***^	76(8.71)	^a^ ***0*.*012*,** ^*b*^ ***3*.*4×10*** ^***−8***^	83(10.23)	^a^0.052,^*b*^ *0*.*118*, ^c^ ***3*.*4×10*** ^***−6***^
**Fasting Plasma Glucose (mmol/L)**	5.01(0.45)	5.55(0.68)	^a^ *0*.*083*	8.09(4.27)	^a^ ***1*.*8×10*** ^***−23***^,^*b*^ ***8*.*8×10*** ^***−12***^	7.79(3.55)	^a^ ***1*.*4×10*** ^***−42***^, ^***b***^ ***3*.*5×10*** ^***−16*,**^ ^c^ *0*.*999*
**Glycosylated A1c (%)**	5.58(0.44)	6.00(0.50)	^a^ *0*.*171*	7.91(2.75)	^a^ ***2*.*7×10*** ^***−23***^, ^*b*^ ***4*.*2×10*** ^***−12***^	8.18(2.04)	^a^ ***1*.*9×10*** ^***−48***^, ^*b*^ ***1*.*2×10*** ^***−22***^, ^c^ *0*.*999*
**Insulin (pmol/L)**	48.8 (38.0)	83.1(51.29)	^a^ ***1*.*8×10*** ^***−8***^	60.8(57.86)	^a^ *0*.*133*_,_ ^*b*^ **0.029**	114(85.11)	^a^ ***5*.*4×10*** ^***−36***^, ^*b*^ ***0*.*001*,** ^c^ ***7*.*7×10*** ^***−11***^
**HOMA-β (%)**	98(40.74)	116(47.86)	^a^ *0*.*518*	48(47.54)	^a^ ***1*.*99×10*** ^***−10***^, ^*b*^ ***1*.*01×10*** ^***−11***^	81(78.72)	^a^ ***0*.*023***, ^*b*^ ***4*.*7×10*** ^***−4***^, ^c^ ***2*.*4×10*** ^***−6***^
**HOMA-IR**	1.04(0.79)	1.81(1.10)	^a^ ***1*.*09×10*** ^***−8***^	1.60(1.58)	^a^ ***9*.*4×10*** ^***−5***^, ^*b*^0.999	2.88(2.57)	^a^ ***1*.*5×10*** ^***−46***^, ^*b*^ ***5*.*6×10*** ^***−7***^, ^c^ ***3*.*3×10*** ^***−9***^
**Total-Cholesterol (mmol/L)**	5.16(1.05)	5.04(1.00)	^a^0.999	4.61(1.15)	^a^ ***0*.*004*,** ^*b*^ *0*.*111*	4.75(1.15)	^a^ ^,^ ***0*.*001***, ^*b*^0.250, ^c^0.999
**High-Density Lipoprotein Cholesterol (mmol/L)**	1.50(0.36)	1.18(0.26)	^a^ ***1*.*2×10*** ^***−12***^	1.36(0.26)	^a^ ***0*.*025*,** ^*b*^ ***0*.*003***	1.11(0.27)	^a^ ***3*.*4×10*** ^***−34***^, ^*b*^ *0*.*335*, ^c^ ***1*.*5×10*** ^***−8***^
**Low-Density Lipoprotein Cholesterol (mmol/L)**	4.19(1.00)	3.37(0.89)	^a^0.300	2.73(1.12)	^a^ ***0*.*002*,** ^*b*^ *0*.*777*	2.75(0.98)	^a^ ***3*.*4×10*** ^***−07***^, ^*b*^0.324,^c^0.999
**Triglycerides (mmol/L)**	1.04(0.58)	1.72(0.71)	^a^ ***7*.*8×10*** ^***−12***^	0.96(0.34)	^a^ *0*.*999*, ^*b*^ ***2*.*5×10*** ^***−10***^	1.75(1.45)	^a^ ***2*.*1×10*** ^***−23***^, ^*b*^ *0*.*999*, ^c^ ***1*.*9×10*** ^***−15***^

The results presented represent geometric means (SD)

^a^ *vs* normal control group

^b^ *vs* non-diabetes MetS group

^c^ *vs* type 2 diabetes mellitus without metabolic syndrome evaluated using ANOVA.

When fasting serum levels of sCD26/DPP-IV and active GLP-1 were evaluated by general linear model, serum levels of sCD26/DPP-IV were significantly higher in T2DM subjects with MetS [1199 (245) ng/mL] than in normal subjects [1089 (281) ng/mL] (*p* = 1.2×10^−4^). Likewise, serum sCD26/DPP-IV levels were significantly higher in T2DM subjects without MetS [1195 (204) ng/mL] than in normal subjects [1089 (281) ng/mL] (*p* = 0.015). On the other hand, serum levels of sCD26/DPP-IV were significantly higher in T2DM subjects with MetS [1199 (245) ng/mL] than in non-diabetic MetS patients [1120 (275) ng/mL] (*p* = 0.041). Additionally, no significant difference in serum sCD26/DPP-IV levels was noted between T2DM patients with or without MetS, or between control and non-diabetic MetS subjects (Table 2).

**Table 2:** Comparison of fasting serum levels of sCD26/DPP-IV and active GLP-1 between normal, non-diabetic metabolic syndrome, type 2 diabetes mellitus subjects with and without metabolic syndrome and total type 2 diabetes mellitus.

	Non-diabetes n = 235			Type 2 diabetes n = 314				Total type 2 diabetes	
Parameters	Normal Control n = 164	MetS n = 71	P-Value	Without MetS n = 57	P-Value	With MetS n = 257	P-Value	Total type 2 diabetes n = 314	P-Value
**sCD26/DPP-IV (ng/mL)**	1089 (281)	1120 (275)	^*a*^ *0*.*427*	1195 (204)	^*a*^ **0.015**, ^b^ *0*.*139*	*1199 (245)*	^*a*^ ***1*.*2×10*** ^***−4***^, ^b^ ***0*.*041*,** ^c^ *0*.*933*	1198 (239)	^*a*^ ***5*.*7×10*** ^***−5***^, ^b^ ***0*.*038***
**Active GLP-1 (pmol/L)**	4.26 (3.89)	3.94 (3.71)	^*a*^ *0*.*340*	3.53 (2.24)	^*a*^ **0.028**, ^b^ *0*.*270*	3.74 (2.29)	^*a*^ ***0*.*020*,** ^b^ *0*.*487*,^c^ *0*.*479*	3.71 (2.28)	^*a*^ ***0*.*010***, ^b^ *0*.*397*

The results presented represent geometric means (SD), adjusted for age, race and gender.

^a^vs control group

^b^vs non-diabetic metabolic syndrome group

^c^vs type 2 diabetes mellitus without metabolic syndrome which evaluated using univariate (General Linear Model).

Bold values are significant. MetS: metabolic syndrome.

In contrast with the noted increase in sCD26/DPP-IV levels, the fasting serum levels of active GLP-1 were significantly lower in T2DM subjects with MetS [3.74 (2.29) pmol/L] than in normal subjects [4.26 (3.89) pmol/L] (*p* = 0.020). Likewise, serum active GLP-1 levels were significantly lower in T2DM subjects without MetS [3.53 (2.24) pmol/L] than in normal subjects [4.26 (3.89) pmol/L] (*p* = 0.028) (Table 2).

Additionally, sCD26/DPP-IV levels were negatively correlated with active GLP-1 levels in both T2DM patients with MetS (r_s_ = -0.324; *p* < 0.001) and T2DM patients without MetS (r_s_ = −0.299; *p* < 0.001) after adjusting for age, gender, and race (Table 3).

**Table 3:** Correlation between of fasting serum levels of sCD26/DPP-IV and active GLP-1 among normal, non-diabetic metabolic syndrome, type 2 diabetes mellitus subjects with and without metabolic syndrome.

Group	r_*s*_	P-value
**Active GLP-1 (pmol/L)**		
**Normal Control n = 164**	-0.198	***0*.*019***
**Non-diabetes MetS n = 71**	-0.139	*0*.*303*
**Type 2 diabetes without MetS n = 57**	-0.299	***< 0*.*001***
**Type 2 diabetes with MetS n = 257**	-0.324	***< 0*.*001***

The results are presented as r_s_ and (P-value) assessed by Spearman partial correlation adjusted for, age, race and gender. Bold values are significant.

### Associations between sCD26/DPP-IV levels, MetS, and A1c levels among T2DM patients

The multiple linear regression analysis (adjusted for age, gender, race, and duration of diabetes) showed that serum levels of sCD26/DPP-IV in diabetic patients were associated with increased A1c levels (B = 19.96, *p* = 0.009), but not associated with insulin resistance (B = 2.44, *p* = 0.525) (Table 4).

**Table 4:** Association of fasting serum levels of sCD26/DPP-IV with MetS and A1c levels among type 2 diabetes patients.

Parameters	B	P-value
**Body Max Index (Kg/m** ^**2**^ **)**	4.64	*0*.*100*
**HOMA- β (%)**	-0.068	*0*.*693*
**Insulin resistance**	2.44	*0*.*525*
**Glycosylated A1c (%)**	19.96	*0*.*009*
**Triglycerides (mmol/L)**	0.492	*0*.*964*
**HDL-cholesterol (mmol/L)**	27.60	*0*.*610*

The results are presented as unstandardized coefficients; B and (P-value) assessed using multiple linear regression adjusted for, age, race, gender, and duration of diabetes. Bold values are significant. B: coefficient for the relationship between the dependent variable “DPP-IV level” and the independent variable “diabetic and metabolic biomarker”. The positive sign of the coefficient implies a direct relationship, and the negative sign implies an inverse relationship.

### Associations between sCD26/DPP-IV levels, MetS, and T2DM parameters among controls

The associations between serum levels of sCD26/DPP-IV, MetS, and T2DM parameters in the control group were investigated by hierarchical linear regression after adjusting for age, race, and gender. This analysis showed that serum levels of sCD26/DPP-IV were associated with increased BMI (B = 0.003, *p* = 0.023), cholesterol (B = 0.001, *p* = 0.001), and LDL-c (B = 0. 001, *p* = 0.001) (Table 5).

**Table 5:** Association of fasting serum levels of sCD26/DPP-IV with diabetic and metabolic parameters among normal subjects.

Parameters	B/r^*2*^	P-value
**Body Mass Index (kg/m** ^**2**^ **)**	**0.003/0.105**	***0*.*023***
**Waist circumference (cm)**	0.006/0.284	*0*.*055*
**Triglyceride (mmol/L)**	0.0003/0.116	*0*.*134*
**Cholesterol (mmol/L)**	0.001/0.107	***0*. *001***
**LDL-cholesterol (mmol/L)**	0.001/0.094	***0*.*001***
**HDL-cholesterol (mmol/L)**	-2.461-5/0.333	*0*.*796*
**Fasting Blood Sugar (mmol/L)**	0.0001/0.077	*0*.*420*
**Glycosylated A1c (%)**	0.0001/0.026	*0*.*531*
**HOMA-β (%)**	0.017/0.053	*0*.*125*
**Insulin resistance**	0.0003/0.070	*0*.*073*

The results are presented as unstandardized coefficients; B, r^2^ and (P-value) assessed using hierarchical linear regression adjusted for, age, race, and gender. Bold values are significant. B: coefficient for the relationship between the dependent variable “metabolic syndrome and T2DM parameters” and the independent variable “DPP-IV level.” The positive sign of the coefficient implies a direct relationship, and the negative sign implies an inverse relationship.

### Associations between sCD26/DPP-IV levels and metformin

Diabetic subjects were divided into 4 subgroups: those receiving monotherapy with metformin (n = 34); those receiving combination therapy without metformin [instead receiving sulfonylurea (SU), and thiazolidinedione (TZD) with or without insulin] (n = 28); those receiving combination therapy with metformin (metformin, SU, and TZD with or without insulin) (n = 241); and non-treated diabetes (n = 8).

The general linear model indicated that patients treated with monotherapy (metformin) had significantly lower serum levels of sCD26/DPP-IV [1048 (275) ng/mL] than patients receiving combination therapy without metformin [1355 (166) ng/mL] (*p* = 2.9×10^−5^). Likewise, patients treated with combination therapy that included metformin had significantly lower serum levels of sCD26/DPP-IV [1205 (229) ng/mL] than patients receiving combination therapy without metformin [1335 (166) ng/mL] (*p* = 0.023) (Table 6).

**Table 6:** Comparison of fasting serum levels of sCD26/DPP-IV between combination therapy without metformin, combination therapy with metformin, monotherapy with metformin and non- treated among subjects with type 2 diabetes.

	* SU +TZD w/wo insulin(n = 28)	Metformin + SU + TZD w/wo insulin (n = 241)	P-Value	Monotherapy with metformin (n = 34)	P-Value	Non-treated (n = 8)	P-Value
Parameter							
**sCD26/DPP-IV (ng/mL)**	1355 (166.0)	1205 (229.1)	^a^ ***0*.*023***	1048 (275.4)	^a^ ***2*.*9×10*** ^***−5***^, ^*b*^ ***0*.*001***	1123 (302.0)	^a^ *0*.*056*, ^*b*^ *0*.*385*,^*c*^ *0*.*432*

The results presented represent geometric means (SD), adjusted for age, gender, and race

^a^vs combination therapy without metformin (SU + TZD with or without insulin)

^b^vs combination therapy with metformin (metformin + SU + TZD with or without insulin)

^c^vs monotherapy with Metformin which evaluated by univariate (General Linear Model).

Bold values are significant.

*Sulfonylurea (SU), Thiazolidinedione (TZD) with or without insulin.

This association was confirmed by multiple linear regression analysis, and the results remained significant only for patients treated with monotherapy that included metformin (B = -201.6, *p* = 0.041), after adjusting for age, race, gender, BMI, A1c, and duration of diabetes (Table 7).

**Table 7:** Association of fasting serum levels of sCD26/DPP-IV with antidiabetes medications groups among type 2 diabetes patients.

Parameters	B	P-value
* **SU+TZD w/wo insulin**	24.0	*0*.*760*
**metformin +SU+ TZD w/wo insulin**	-131.6	*0*.*120*
**Monotherapy with Metformin**	-201.6	***0*.*041***
**Non-treated**	-97.4	*0*.*085*

The results are presented as unstandardized coefficients; B and (P-value) assessed using multiple linear regression adjusted for, age, race, gender, BMI, HbA1c, and duration of diabetes. Bold values are significant. B: coefficient for the relationship between the dependent variable “DPP-IV level” and the independent variable “diabetic drugs”. The positive sign of the coefficient implies a direct relationship, and the negative sign implies an inverse relationship.

*Sulfonylurea (SU), Thiazolidinedione (TZD) with or without insulin.

## Discussion

In this study, the observed fasting serum levels of sCD26/DPP-IV in T2DM patients were higher than that in normal subjects; these results are in agreement with findings by Lee et al., who excluded patients treated with metformin and/or thiazolidinedione therapy [17]. These results are in contrast to findings by Meneilly et al. and Korosi et al. [26, 27], who reported decreased sCD26/DPP-IV levels in diabetic patients.

There are several studies that support increased DPP-IV activity in T2DM patients [23, 28]; however, the cause for the increase in DPP-IV activity in diabetic patients remains unclear. Pala et al. [29] indicated that human glomerular endothelial cells that are exposed to high concentrations of glucose promote the biosynthesis of DPP-IV in vitro. In addition, another study by Pala et al. [30] reported that DPP-IV activation was not induced in control subjects, T2DM patients, or patients with impaired glucose tolerance according to oral glucose loading. Similarly, research by Ryskjaer et al. [31] indicated that DPP-IV activity was increased in T2DM patients. However, observed DPP-IV activity was not altered after meal ingestion and subsequent acute changes in plasma glucose. Recently, Aso et al. demonstrated that sCD26/DPP-IV levels in healthy subjects exhibited an acute increase after oral glucose loading, and this abrupt increase may be associated with the presence of nonalcoholic fatty liver and/or insulin resistance [32]. According to these findings, it is believed that DPP-IV biosynthesis is associated with long-term exposure to high levels of glucose.

Interestingly, in line with the observed increased sCD26/DPP-IV levels and decreased active GLP-1 levels in T2DM subjects, sCD26/DPP-IV levels showed a negative correlation with active GLP-1 levels in T2DM patients both with and without MetS. In Chinese subjects with T2DM, it was observed that GLP-1 levels were lower than in the normal glucose tolerance subjects [33]. A similar finding in Caucasian subjects indicated that GLP-1 levels decreased significantly in T2DM patients than in the control subjects [34]. Even in patients with T1DM, GLP-1 levels were lower than in healthy subjects [35]. Overall, in subjects with T2DM, the significant reduction in the incretin effect has been attributed primarily to decreased circulating levels of GLP-1, which may be secondary to either increased degradation by DPP-IV or its decreased secretion by the gut [36]. Pala et al. suggested that the decrease in GLP-1 levels in the early stage of the disease is the most dominant because of impairment in its secretion. However, increased sCD26/DPP-IV activity has a major role after the disease has been in place for longer durations [30].

This study also demonstrated that higher serum levels of sCD26/DPP-IV in T2DM patients were associated with increased A1c, which is in agreement with findings by Lee et al. [17]. On the other hand, it has been reported that treatment with DPP-IV inhibitors may improve A1c levels in T2DM patients [37]. In addition, previous studies [31,38] have demonstrated that DPP-IV activity showed significant correlations with serum A1c levels in diabetic patients. However, these results differ from recently published research by Fadini et al. [23].

We found that serum levels of sCD26/DPP-IV in diabetic patients were not associated with insulin resistance (HOMA-IR). Such findings were in contrast to the findings of Lee et al. [17], which could be due to differences in treatment profiles, as these may affect insulin resistance. Associations between sCD26/DPP-IV levels and MetS parameters were assessed in the control group, since non-diabetic subjects with MetS were under treatment. Research by Lamers et al. [5] demonstrated that adipose tissue inflammation and enlargement of adipocytes enhances the release of soluble DPP-IV from fat cells into the circulation. Furthermore, other studies [5, 23] have reported that circulating DPP-IV correlated with different markers for MetS, including plasma TG, BMI, and waist circumference. Recently, Yang et al. demonstrated that increased DPP-IV activity in healthy Chinese could independently predict MetS, insulin resistance [15], and the risk of developing hypertension [39]. Our current study showed that increased serum levels of sCD26/DPP-IV were associated with increased BMI, total cholesterol, and LDL-c. Our research provides evidence that sCD26/DPP-IV may be useful as a biomarker for increased risk of obesity or metabolic syndrome.

Our findings are also consistent with a previous study that suggested that metformin reduced serum sCD26/DPP-IV levels [17]. We found lower sCD26/DPP-IV levels in diabetic patients on monotherapy (e.g., metformin). Numerous studies [23, 40, 41] have demonstrated that sCD26/DPP-IV activity in metformin users was lower. Some studies have suggested that metformin lowered DPP-IV activity by repressing the release of its soluble isoforms from cells [41, 42]. On the other hand, metformin was also postulated to lower plasma DPP-IV activity indirectly through upregulation of GLP-1 receptors’ expression in pancreatic β-cells and increasing plasma GLP-1 levels [43].

## Conclusion

Our results demonstrated that fasting serum levels of sCD26/DPP-IV were increased inT2DM patients both with MetS and without MetS. In contrast, active GLP-1 levels were decreased in T2DM patients both with and without MetS. In addition, sCD26/DPP-IV levels were associated with A1c and were negatively correlated with active GLP-1 levels. However, sCD26/DPP-IV levels were decreased in patients on monotherapy that included metformin.

In control subjects, sCD26/DPP-IV levels were found to be associated with increased BMI, cholesterol, and LDL-c cholesterol. Further studies are necessary to explore the reasons for increased sCD26/DPP-IV levels in T2DM patients and to ascertain whether sCD26/DPP-IV level is an early marker for MetS and/or T2DM.
